# From kelp forests to turf reefs: Patterns, drivers, and impacts to functional diversity

**DOI:** 10.1002/ecy.70408

**Published:** 2026-05-17

**Authors:** Shane P. Farrell, Dara S. Yiu, Stuart K. Ryan, Rene D. Francolini, Courtney E. Stuart, Jonathan S. Lefcheck, Yasmina M. Shah Esmaeili, Douglas B. Rasher

**Affiliations:** ^1^ Bigelow Laboratory for Ocean Sciences East Boothbay Maine USA; ^2^ School of Marine Science University of Maine Walpole Maine USA; ^3^ School of Geography and the Environment University of Oxford Oxford UK; ^4^ University of Maryland Center for Environmental Science Cambridge Maryland USA

**Keywords:** climate change, functional traits, phase shift, resilience, state shift, turf algae

## Abstract

Kelp forests are declining in many regions due to ocean warming, predator loss, and other anthropogenic stressors. In areas of rapid ocean warming, including the southern Gulf of Maine, these ecosystems have transitioned to a novel state dominated by low‐lying mats of turf algae. However, the pace, drivers, and ecological consequences of this transition remain unclear. Here, we used field surveys from 32 sites over 5 years (2018–2023) to reveal a continuation of kelp forest collapse and northward expansion of turf algae across Maine's coast. Next, we united data on benthic cover with key environmental variables in a structural equation model to show that turf algae were directly enhanced by higher ocean temperatures and decreased wave disturbance and indirectly enhanced by a warming‐induced loss of kelp cover. Lastly, we revealed the shift from kelp to turf yielded a seaweed assemblage dominated by traits associated with rapid growth, high surface area‐to‐volume ratios, and markedly reduced canopy height, indicating declines in habitat provisioning and carbon storage with kelp forest loss. Our findings highlight the accelerating impacts of climate change on temperate reef ecosystems and the vital services they provide. Further, we provide insights into a new state shift that is now occurring globally and underscore the need for urgent actions to mitigate further loss of foundational kelp forests.

## INTRODUCTION

A state shift refers to a rapid and lasting transformation in an ecosystem's structure and function (Scheffer, 2009; Scheffer et al., 2001; Scheffer & Carpenter, 2003). Unlike temporary ecosystem fluctuations, state shifts cross thresholds that generate self‐reinforcing feedbacks, making such shifts difficult to reverse (Connell & Sousa, 1983; Holling, 1973; Sutherland, 1974). Human‐induced stressors are generally the drivers of state shifts, and have amplified the frequency and severity of these shifts in both terrestrial ecosystems (e.g., savanna‐to‐grassland, woodland‐to‐boreal) and marine ecosystems (e.g., coral‐to‐macroalgae, saltmarsh‐to‐mudflat, kelp forest‐to‐urchin barren) (Ling et al., 2015; Scheffer, 2009; Scheffer et al., 2001). However, even with a long history of investigation into state shifts (Conversi et al., 2015), climate change has made this phenomenon increasingly prevalent and unpredictable, often triggering “ecological surprises” (Filbee‐Dexter et al., 2017) and the emergence of novel ecosystem states that are poorly understood and thus difficult to manage for recovery.

Kelp forests are globally important ecosystems, creating structural habitat and food resources for myriad species (Duggins, 1980; Duggins et al., 1989, 1990; Krause‐Jensen & Duarte, 2016; Miller et al., 2018; Steneck et al., 2002; Teagle et al., 2017; Weigel & Pfister, 2019) while also providing ecosystem services valued globally at billions of dollars per year (Eger et al., 2023). Nevertheless, kelp forests are threatened by regional and global stressors (Krumhansl et al., 2016) that have given rise to state shifts, including predator loss and a resultant trophic cascade leading to overgrazing by sea urchins (Filbee‐Dexter & Scheibling, 2014; Ling et al., 2009, 2015). Recently, a new type of state shift has emerged in areas of rapid ocean warming, where kelp forests have given way to low‐lying carpets of filamentous “turf” algae (Filbee‐Dexter & Wernberg, 2018).

Kelp‐to‐turf state shifts were recently observed in Australia (Wernberg et al., 2013), Japan (Pessarrodona et al., 2021), the Mediterranean Sea (Filbee‐Dexter & Wernberg, 2018), the Baltic Sea (Eriksson et al., 2002), and the Northeast (Moy & Christie, 2012) and Northwest Atlantic Oceans (Feehan et al., 2019; Suskiewicz et al., 2024), and have been linked to significant declines in ecosystem function and resilience (e.g., Airoldi, 2003; Farrell et al., 2025; Feehan et al., 2019; O'Brien et al., 2015; Yiu et al., 2025). Yet, while the general drivers of kelp forest decline are well known around the globe (Filbee‐Dexter & Wernberg, 2018; Smale, 2020; Suskiewicz et al., 2024), the factors that govern turf algae abundance, and the identity and diversity of turf algae species involved in associated state shifts, have not yet been rigorously quantified. Furthermore, while the kelp‐to‐turf transition is known to impact primary production (Pessarrodona, Assis, et al., 2022; Pessarrodona, Vergés, et al., 2022), secondary production (Pessarrodona, Assis, et al., 2022; Pessarrodona, Vergés, et al., 2022), predator–prey interactions (Dijkstra et al., 2019; O'Brien et al., 2018; Pessarrodona et al., 2021), nutrient dynamics (Ramsay‐Newton et al., 2017), and reef biodiversity (Dijkstra et al., 2017; Wernberg et al., 2013), we lack an understanding of how this novel state shift alters the “functional traits” possessed by the macroalgal community and ultimately how shifts in trait composition will translate to changes in ecosystem function.

**FIGURE 1:**
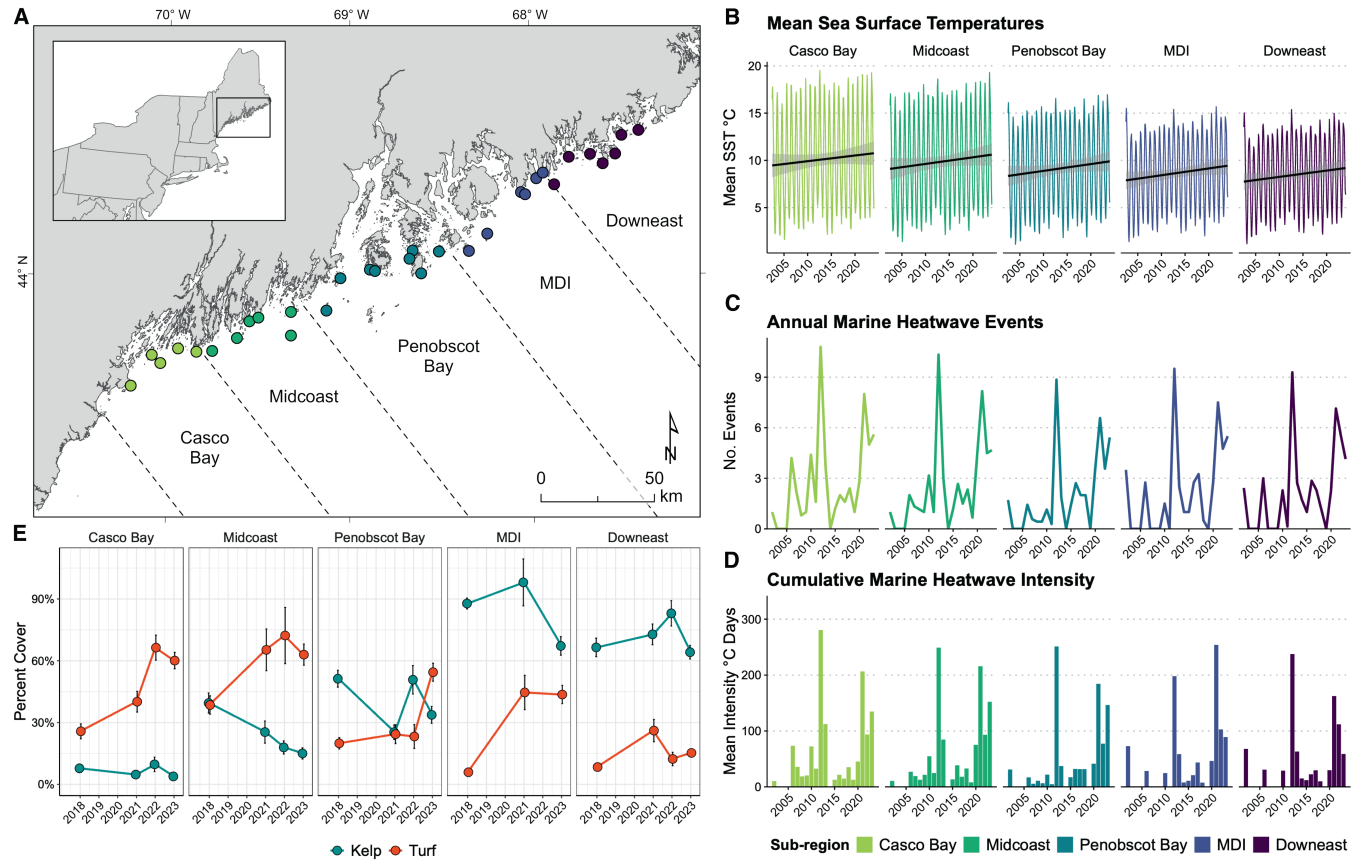
(A) Map of study sites. (B) Subregional daily mean sea surface temperature (2002–2024). (C) Annual number of marine heat wave (MHW) events per subregion (≥5 consecutive days above the seasonally varying 90th percentile). (D) Annual cumulative MHW intensity, summed across events in degrees Celsius·days. (E) Changes in kelp (blue dots) and turf algae (red dots) abundance (percent cover) on outer coastal reefs, from 2018 to 2023. Each point denotes a subregional mean and error bars represent SE. Note gap between 2018 and 2021.

Here, we address these knowledge gaps in the Gulf of Maine, a rapidly warming ocean basin that is now experiencing a kelp‐to‐turf transition (Suskiewicz et al., 2024) and thus represents a powerful natural laboratory for studying this global phenomenon. Historically, kelp forests in the Gulf of Maine were structured by top‐down forcing by large predatory fishes such as cod and wolffish, which suppressed herbivorous green sea urchins therein, allowing kelp to dominate throughout the region (Bourque et al., 2008; Steneck & Wahle, 2013). Industrial fishing in the 20th century caused the collapse of predatory groundfish stocks and triggered a state shift to urchin‐dominated reefs devoid of macroalgae (Steneck et al., 2004, 2013), but a sea urchin fishery peaking in the 1990s then drove sea urchins to low numbers, which allowed kelp to recover across the region at the turn of the 21st century (Suskiewicz et al., 2024). Over the last two decades, however, seawater temperatures have risen rapidly in the Gulf of Maine—making it one of the fastest warming marine ecosystems in the world—and marine heat waves (MHWs) have become more frequent and intense (Pershing et al., 2015; Suskiewicz et al., 2024; Figure 1). Moreover, the Maine coast contains a thermal gradient from north to south (a 6°C differential in summer) owing to its coastal oceanography, placing Maine's southern kelp forests in much warmer conditions. Consequently, unusually warm spring and summer seawater temperatures triggered the collapse of kelp forests in southern Maine from 2001 to 2018 (Suskiewicz et al., 2024), and these southern reefs quickly transitioned to turf dominance (Dijkstra et al., 2017; Suskiewicz et al., 2024). Even in northeastern Maine, where conditions are cooler, this warming caused gradual declines in kelp abundance from 2001 to 2018, raising concern that continued warming and more frequent and intense heat waves will further erode reef resilience and associated ecosystem services.

Therefore, to assess the current pace and progression of this novel state shift, and identify the species involved, we conducted surveys at up to 32 sites spanning >250 km of Maine's coastline in 2018 (from Suskiewicz et al., 2024), 2021, 2022, and 2023 and documented turf dynamics at a species‐level resolution. Then, to identify the direct and indirect drivers governing the recent spread of turf algae, we constructed a structural equation model (SEM) incorporating site‐level abiotic data (temperature, wave height) and biotic data (kelp abundance) to assess which factors were causally linked to turf abundance through space and time. Lastly, to understand how this transition alters the net ecological potential of the seaweed community, we compiled species‐specific “functional trait” data to reveal which traits—that is, attributes that underpin ecosystem functioning (Cappelatti et al., 2019; Griffin et al., 2023; Mauffrey et al., 2020)—are gained versus lost from the seaweed community as kelp forests are replaced by turf algae. With these approaches, our study revealed that the kelp‐to‐turf transition is rapidly progressing across the Gulf of Maine in response to climate change. Further, it sheds new light on the direct and indirect drivers of state shifts on temperate reefs and increases our understanding of the potential functional consequences.

## METHODS

### Site characteristics and study design

For this study, we divided the Maine coast into five subregions based on hydrography, geographic features, usage, and prior publications (McManus et al., 2023; Suskiewicz et al., 2024). From southwest to northeast, these are Casco Bay, Midcoast, Penobscot Bay, Mount Desert Island (MDI), and Downeast (Figure 1a). Casco Bay is an embayment from Cape Elizabeth to Cape Small and Penobscot Bay is a similarly well‐defined embayment that spans from Port Clyde to Isle au Haut. The Midcoast and MDI subregions are interspersed between these, while Downeast stretches from the Schoodic Peninsula to the United States‐Canada Border. These divisions also broadly align with state fishery management zones (e.g., MDI and Downeast correspond to designated sea urchin Zones 1 and 2, respectively).

We selected study sites within these subregions that were wave‐exposed locations on the outer coast (i.e., ends of peninsulas or coastal islands) that were characterized by hard bottom (boulder or ledge) habitat at 5–10 m depth (mean lower low water, MLLW). These sites supported kelp forests in the past and have a known ecological history due to long‐term monitoring (Suskiewicz et al., 2024). Moreover, we focused on sites that were dispersed at regular intervals (~2–15 km apart) and spanned most of Maine's coastline (> 250 km linear distance) making them a representative sample of the region (see Appendix S1: Table S1 for site coordinates and all other data can be found in Farrell et al., 2026).

While some study sites were revisited in multiple years of sampling (2018, 2021–2023), others were not (Appendix S1: Table S1), resulting in variation in the number of sites studied per year in each subregion—Casco Bay (3–5 sites/year), Midcoast (2–5 sites/year), Penobscot Bay (3–6 sites/year), MDI (2–5 sites/year, except in 2022 when it was not surveyed), Downeast (3–6 sites/year). Likewise, there was variation in the total number of sites studied per year (2018 = 25 sites, 2021 = 16 sites, 2022 = 11 sites, 2023 = 27 sites; a total of 32 unique sites). Nevertheless, the survey design provided sufficient power and replication to robustly analyze the status and trends of rocky reefs across the region. We note that the 2018 data were previously published in Suskiewicz et al. (2024) and were incorporated into this analysis for continuity with the previous investigation.

### Macroalgal percent cover and biomass

We documented the potential spread of turf algae across Maine's coast and characterized reef species composition over a 5‐year period (Figure 1a). Each year, we surveyed sites between July and September (i.e., during oceanographic summer), using identical methods. At each site, we deployed (via SCUBA) a 40‐m tape set perpendicular to shore along the 5–7 m depth isobath (MLLW). Within replicate 1‐m^2^ quadrats deployed at set intervals along the tape (*n* = 8 quadrats per site), we estimated the percent cover of all canopy‐forming kelp species. Within a 0.25‐m^2^ area of each quadrat, we estimated the percent cover of all non‐canopy‐forming species (including bladed, foliose, and filamentous or uniseriate algae). Percent cover estimates were binned as follows: 0%, 1%, 5%, 10%, 20%, 30%, 40%, 50%, 60%, 70%, 80%, 90%, 95%, 99%, 100%. We used this semilogarithmic, binned scale because it (1) reduces observer error and avoids pseudo‐precision in visual estimates—especially at high cover—while still capturing rare taxa at low covers (Dethier et al., 1993); (2) follows long‐standing vegetation survey practice that prioritizes finer resolution for low cover and high cover classes (Ellenberg & Mueller‐Dombois, 1974); and (3) yields data well‐suited to community analyses (Clarke et al., 2001). Here, we consider turf algae to be filamentous (multiseriate or uniseriate) species that create highly branched thalli, clumps, or mats and are 0.5–15 cm in height. Of note, this definition does not include foliose or bladed macroalgae (which some studies have considered as turf species; Pessarrodona et al., 2021). We adhered to a stricter definition (see Connell et al., 2014) that only includes filamentous species.

To define the biomass and species/genus‐level identity of turf algae (which can be difficult to discern underwater), at each site we harvested all canopy‐forming kelps from within 1‐m^2^ quadrats (*n* = 3–6 per site) as well as all non‐canopy‐forming (“understory”) seaweed species from a 0.25‐m^2^ area within each quadrat (*n* = 3–6 per site). These algae were kept cool and brought back to the lab within 6 h, where we identified them to species (via microscopy, where necessary), spun them (20 revolutions in a salad spinner), and weighed them to quantify their biomass and relative abundance.

### In situ and satellite environmental data

To understand the environmental drivers of turf spread, we collected temperature and wave height data from a combination of benthic in situ sensors and satellite‐derived data products. We deployed ocean wave height loggers (OWHLs; Lyman et al., 2020) at 5–7 m depth on the reef (MLLW) at a subset of sites, from May to September, in 2021, 2022, and 2023. Significant wave height (SWH) (pressure) was recorded four times per second. Pressure was converted to SWH using the *owhlR* package (0.2.0; Miller, 2020) in R (R Core Team, 2024).

To obtain year‐round estimates of SWH for all sites and years, we corrected remotely sensed data layers using our in situ measurements. SWH was obtained from the European Center for Medium‐Range Weather Forecasts' (ECMWF) Reanalysis 5 (ERA5) dataset (0.5° × 0.5° horizontal resolution) on an hourly basis through the Climate Data Store (Hersbach et al., 2020). While the resolution of the satellite wave data (0.5° × 0.5°) is relatively coarse, we used linear models to correct these values based on our in situ measurements. We chose this option as a balance between the logistical constraints of measuring in situ wave height at 32 sites over multiple years versus the coarse resolution associated with only using satellite‐derived data (to our knowledge the ERA5 dataset is the finest resolution for the Gulf of Maine). SWH models were constructed by splitting data into test and train sets to ensure accurate model predictions.

Daily mean sea surface temperatures for the last 22 years were obtained from NASA's Multi‐scale Ultra‐high Resolution (MUR) Sea Surface Temperature (SST) dataset (0.01° × 0.01° horizontal resolution grid coordinates) using the *rerddap* package in R (version 1.2.1, Chamberlain et al., 2025). Using the long‐term SST data, we detected MHWs following Hobday et al. (2016), which entail warming events that run ≥5 consecutive days where daily SST exceeds the seasonally varying 90th percentile threshold. The analysis was implemented using *heatwaveR* (0.5.4; Schlegel & Smit, 2018). Because a 30‐year baseline is not available at 1 km resolution, we set the climatology period to the full record for each subregion (2002–2024; to allow for the longest climatology period possible), acknowledging that a shorter baseline can shift the percentile thresholds.

### Statistical analysis: Changes in kelp and turf abundance over time and space

We employed generalized linear mixed‐effects models (GLMMs) to understand changes in turf algae and kelp abundance (which were scaled and bound between 0 and 1) from 2018 to 2023. We constructed two individual beta regression models—one for changes in total turf percent cover, and one for changes in total kelp percent cover—with a logit link function, using the *glmmTMB* package (1.1.10; Brooks et al., 2017) in R (Appendix S1: Tables S2 and S3).

*Kelp Model*

$$P\left({\mathrm{Kelp}}_{ijt}=0\right)=\pi_{ijt}$$

$${\mathrm{Kelp}}_{ijt}|{\mathrm{Kelp}}_{ijt}>0\sim Beta(\mu_{ijt}\phi_{ijt},(1-\mu_{ijt})\phi_{ijt})$$

$$\mathrm{logit}\left(\mu_{\mathrm{ijt}}\right)=\beta_{0}+\beta_{1}\text{Year}+\beta_{2}\text{Subregion}\;\;+\beta_{3}\left(\text{Year}\times \text{Subregion}\right)+\alpha_{\text{Site}\left(i\right)}+\alpha_{\mathrm{Obs}\left(\mathrm{ijt}\right)}$$

$$\mathrm{logit}\left(\pi_{\mathrm{ijt}}\right)=\gamma_{0}+\gamma_{1}\text{Year}+\gamma_{2}\text{Subregion}$$

$$\log\left(\phi_{\mathrm{ijt}}\right)=\delta_{0}+\delta_{1}\text{Year}+\delta_{2}\text{Subregion}+\delta_{3j}\left(\text{Year}\times \text{Subregion}\right)$$

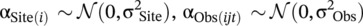




*Turf Model*

$$P\left({\mathrm{Turf}}_{ijt}=0\right)=\pi_{ijt}$$

$${\mathrm{Turf}}_{ijt}|{\mathrm{Turf}}_{ijt}>0\sim Beta(\mu_{ijt}\phi_{ijt},(1-\mu_{ijt})\phi_{ijt})$$

$$\mathrm{logit}\left({\text{μ}}_{\mathrm{ijt}}\right)=\beta_{0}+\beta_{1}\text{Year}+\beta_{2}\text{Subregion}\;\;+\beta_{3}\left(\text{Year}\times \text{Subregion}\right)+\alpha_{\text{Site}\left(i\right)}+\alpha_{\mathrm{Obs}\left(\mathrm{ijt}\right)}$$

$$\mathrm{logit}\left(\pi_{\mathrm{ijt}}\right)=\gamma_{0}+\gamma_{1}\text{Year}+\gamma_{2}\text{Subregion}$$

$$\log\left(\phi_{\mathrm{ijt}}\right)=\delta_{0}+\delta_{1}\text{Year}+\delta_{2}\text{Subregion}$$

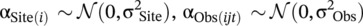

where Kelp_*ijt*_ and Turf_*ijt*_ are the percent cover (of either kelp or turf) in site (*i*) in subregion (*j*) in year (*t*), β_0_ is the intercept, and β_1_, β_2_, and β_3_ represent the fixed effects of year, subregion, and their interaction respectively. α_Site(*i*)_ is the random intercept for site and α_Obs(*ijt*)_ is the observation‐level random effect to account for overdispersion. The beta distribution includes two additional parameters: pi ($\pi_{\mathrm{ijt}}$), which is the zero‐inflation probability of predicting the chance of observing a true zero rather than a beta‐distributed value, and phi ($\phi_{\mathrm{ijt}}$), which is the precision parameter describing the inverse of the variance of the beta distribution. We allowed phi ($\phi_{\mathrm{ijt}}$) to vary as a function of year and subregion to accommodate heteroskedasticity among space and time, rather than assuming constant variance across all observations. Models were constructed with and without interactions between year and subregion for the conditional, zero‐inflated, and dispersion components and compared using Akaike information criterion (AIC) and Bayesian information criterion (BIC). Models with the lowest AIC are presented here. Model evaluation included checking for overdispersion and assessing the fit of the zero‐inflation component, as well as model assumptions for uniform quantile residuals using the *DHARMa* package (0.4.7; Hartig, 2024).

We quantified temporal trends within each subregion using estimated marginal trends from the fitted models with the *emmeans* package in R (2.0.0; Lenth et al., 2025). For each subregion, we obtained the annual slope for year on the model's link scale, holding other categorical predictors at their reference values and continuous predictors at their population mean. We compared subregional slopes with Tukey's adjusted pairwise contrasts (Appendix S1: Figures S1 and S2; Tables S4 and S5). We held an experiment‐wide α = 0.05, except where noted, to address issues arising from multiple testing.

### Statistical analysis: Turf species richness

We analyzed site‐level turf species richness with a GLMM in *glmmTMB*, using the Conway–Maxwell–Poisson (COM‐Poisson) family with a log link. We included a fixed effect of subregion and random effects for year and site. Model evaluation included checking for overdispersion and assumptions for uniform quantile residuals using *DHARMa* (Appendix S1: Table S6) (Hartig, 2024). Estimated marginal means for each subregion were obtained with *emmeans* and are reported on the response (count) scale with 95% CIs. We calculated pairwise subregion contrasts with a Tukey's multiplicity adjustment (Appendix S1: Table S7).

### Statistical analysis: Identifying drivers of turf spread using SEM

To evaluate the environmental and ecological drivers of turf algae dynamics through space and time, we developed a SEM to evaluate direct and indirect drivers of turf spread. This model included kelp cover as a potential driver of turf abundance, to reflect kelp–turf competition that can manifest through a variety of mechanisms (e.g., space preemption, whiplash effects, shading; Duggins et al., 1990; Irving & Connell, 2006; Kennelly, 1987; Santelices & Ojeda, 1984; Smale et al., 2020; Toohey, 2007). We also included the annual mean SST from the year prior to the survey, given its demonstrated negative effects on kelp in this system (Suskiewicz et al., 2024) as well as its known influence on turf algae growth and reproduction in other regions (Bischoff & Wiencke, 1993; Bjærke & Rueness, 2004; Breeman et al., 1988; Maggs & Stegenga, 1998). Further, we included SWH in the SEM, given the ability of kelp to clear substrates via “whiplash” under high wave intensity (Duggins et al., 1990; Smale et al., 2020) and the ability of turf algae to reproduce asexually through fragmentation in wave swept areas, leading to spore/fragment dispersal (Cecere et al., 2011; Husa & Sjøtun, 2006). While we considered incorporating indicators of MHWs in the SEM directly, we felt the mean SST captured both periods of excessively high temperatures (heat waves) as well as anomalously low temperatures, which may also impact turf abundance. We also considered adding sea urchin density to the model, but sea urchins were largely absent from most sites during the survey period and, when present, occurred in very low densities (Suskiewicz et al., 2024; unpublished data), so we elected to not include this parameter.

We modeled the above relationships with GLMMs using the *nlme* package (3.1‐168; Pinheiro et al., 2025). In each model, we included a random intercept of year nested within site within subregion to account for spatial and temporal autocorrelation, and a continuous autoregression *CAR*(1) correlation structure with the same specification to further account for temporal autocorrelation. We included log transformations where necessary to meet the assumptions of the models (i.e., homogeneity of variance, independence of errors). We inspected residual plots for each submodel, assessed multicollinearity with variance inflation factors (VIFs), and tested for any residual spatial autocorrelation with Moran's *I*. All tests revealed adequate fits with no substantial residual collinearity or autocorrelation.

Recently, Suskiewicz et al. (2024) and Byrnes and Dee (2025) identified a significant issue where correlations between fixed and random effects, such as temperature and site, can introduce omitted variable bias and therefore confound causal interpretation. To address this issue, we used a group‐mean‐centered approach to break the correlation between the fixed and random effects (following Suskiewicz et al., 2024). For each predictor, we calculated: (1) a subregional anomaly term (i.e., each observation minus the long‐term mean of the subregion) and (2) the subregional mean itself. We then added these predictors to each submodel such that the full SEM is captured in the following models:

*Submodel 1*


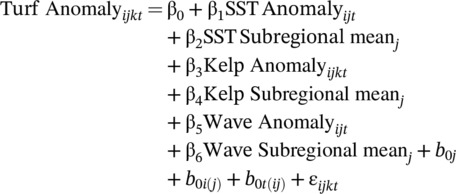

$$b_{0j}\sim N\left(0,{\tau^{2}}_{j}\right),\;b_{0i\left(j\right)}\sim N\left(0,{\tau^{2}}_{i}\right),\;b_{0t\left(ij\right)}\sim N\left(0,{\tau^{2}}_{t}\right),\;\varepsilon_{ijkt}\sim N\left(0,\sigma^{2}\right)$$

$$cor\left(\varepsilon_{ijkt},\varepsilon_{ijkt'}\right)=\rho^{\left|t‐t'\right|}$$

*Submodel 2*


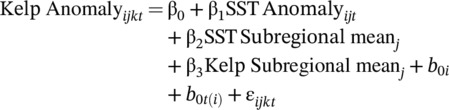

$$b_{0i}\sim N\left(0,{\tau^{2}}_{i}\right),\;b_{0t\left(i\right)}\sim N\left(0,{\tau^{2}}_{t}\right),\;\varepsilon_{ijkt}\sim N\left(0,\sigma^{2}\right)$$

$$cor\left(\varepsilon_{ijkt},\varepsilon_{ijkt'}\right)=\rho^{\left|t‐t'\right|}$$

where (1) Submodel 1 predicted log‐transformed turf percent cover from the within‐site (*i*) observation (*k*) in region (*j*) in year (*t*) of the kelp anomaly, SST anomaly, wave anomaly, and corresponding subregion means; and (2) Submodel 2 predicted the kelp anomaly from the SST anomaly, SST subregion mean, and kelp subregion mean. The SEM was fit and evaluated with the *piecewiseSEM* package (2.3.1; Lefcheck, 2016). While wave disturbance can play a role in kelp resilience (Berry et al., 2021; Starko et al., 2019), we chose not to model the relationship between SWH (wave energy) and kelp cover to allow us to assess the overall goodness of fit of the model using tests of directed separation (Shipley, 2013). The resulting Fisher's *C* statistic was compared to a χ^2^ distribution with 2 df to obtain a model‐wide *p* value. Here, a good fitting model will have *p* > 0.05, indicating no significant discrepancy between the specified model structure and empirical relationships implied by the data (Appendix S1: Tables S8–S10).

### Seaweed functional trait measurements

To reveal how the kelp‐to‐turf transition impacts the functional diversity of the seaweed assemblage, we collated functional trait information for most species found in our biomass collections (Griffin et al., 2023). Trait information was obtained from a database in Mauffrey et al. (2020) and for 16 species not in the database, we measured their traits during the summer of 2023 (for trait measurement metadata, see Appendix S1: Table S11). We focused on seven traits with known links to ecosystem functioning (Cappelatti et al., 2019; Mauffrey et al., 2020): algal branching order, thallus dry matter content, thallus thickness, surface area‐to‐volume ratio, specific thallus area, carbon‐to‐nitrogen (C:N) ratio, and maximum length. These traits underpin photosynthetic ability, capacity to produce large, three‐dimensional structures (vs. small, fine‐scale interstitial complexity), and palatability to consumers. When considered in aggregate, they dictate the net ecological potential of the community (Cappelatti et al., 2019; Mauffrey et al., 2020). Details on measurement methods can be found in Appendix S1: Section S1.

### Statistical analysis: Consequences of turf proliferation on species and functional diversity

To assess how kelp decline and turf proliferation influences algal community diversity, we calculated Simpson's diversity indices on a site‐level basis and modeled them as a function of local state shift dynamics (i.e., the ratio of kelp cover to kelp + turf cover; hereafter the kelp‐to‐turf metric) which was also calculated at the site level. Given that Simpson's diversity (*D*) does not scale linearly, we transformed the index using a Jost transformation: 1/1 − *D*, offering a more straightforward and linear interpretation (i.e., effective number of taxa if all taxa were equivalently abundant). We fit a generalized additive model (GAM) using restricted maximum likelihood in the *mcgv* package (1.9.4; Wood, 2011) as follows:

$${\text{Simpson diversity}}_{i}=N\left(\mu_{i},\sigma^{2}\right)$$

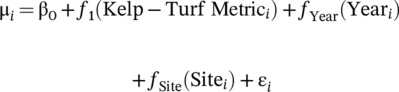

where β₀ is the intercept, f₁ is the smooth function capturing the effect of the kelp‐to‐turf metric, and $f_{\text{Year}}$ and $f_{\text{Site}}$ represent random effects for site and year, respectively. We evaluated model performance through diagnostic checks, such as residual plots, concurvity assessments, and smooth term evaluation.

### Statistical analysis: Algal functional traits

We conducted community functional diversity analyses considering five metrics of functional diversity. These metrics included Rao's quadratic entropy (Rao's *Q*), functional richness, functional evenness, functional dispersion, functional divergence, and the community weighted means of the seven traits measured (Villéger et al., 2008).

To investigate the relationship between the kelp‐to‐turf metric and functional diversity, we fitted a GAM using REML and included smooth terms for the kelp‐to‐turf metric, year (as a random effect), and site (as a random effect). The model was implemented in the *mgcv* package in R, as follows:

$${\text{Functional diversity}}_{i}=N\left(\mu_{i},\sigma^{2}\right)$$

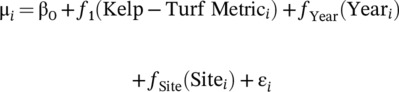




We evaluated each model's performance through diagnostic checks, such as residual plots, concurvity assessments, and smooth term evaluation. We used the same model structure for all functional diversity metrics and the seven community weighted means.

## RESULTS

### Rising sea surface temperatures and MHWs

Daily sea surface temperatures increased over time, suggesting basin‐wide warming even in areas that were historically cooler (e.g., Downeast) (Figure 1b). MHWs were identified in every subregion (Figure 1c), with substantial interannual variability. MHWs occurred one to several times per year—especially during the 2010s and early 2020s—concurrent with the overall trend of warming. Cumulative MHW intensity (Figure 1d) also varied among years within each subregion, with a handful of high‐intensity years in each subregion punctuating otherwise moderate occurrences. MHWs were generally more intense in the south (Casco Bay) than in the north (Downeast); Casco Bay experienced multiple years showing large MHW totals, whereas Downeast usually remained low with occasional moderate spikes. Previous research causally linked these temperature anomalies over the last 20 years to a decline of kelp across the region, as well as the collapse of kelp forests and rise of turf algae in Maine's most southern subregions (Suskiewicz et al., 2024).

### Macroalgal percent cover

Continued subtidal monitoring revealed that a shift from kelp to turf dominance rapidly progressed across Maine's outer coast within just the 5‐year study period. In Casco Bay, which had already become turf‐dominated by 2018 (Suskiewicz et al., 2024), kelp remained rare. From 2018 to 2023, kelp cover further declined in the Midcoast (from 39% to 15%), Penobscot Bay (from 51% to 33%), and MDI (from 88% to 67%) subregions (Figure 1e). During the same period, turf cover increased from 39% to 63% in Midcoast, from 20% to 54% in Penobscot Bay, and from 6% to 44% in MDI (Figure 1e). Conversely, in the most northern, coolest subregion (Downeast), kelp cover did not significantly change and turf algae remained rare (Figure 1e). Thus, nearly half of Maine's outer coast has experienced, or is undergoing, a novel state shift.

### Macroalgal diversity, identity, and relative abundance

**FIGURE 2:**
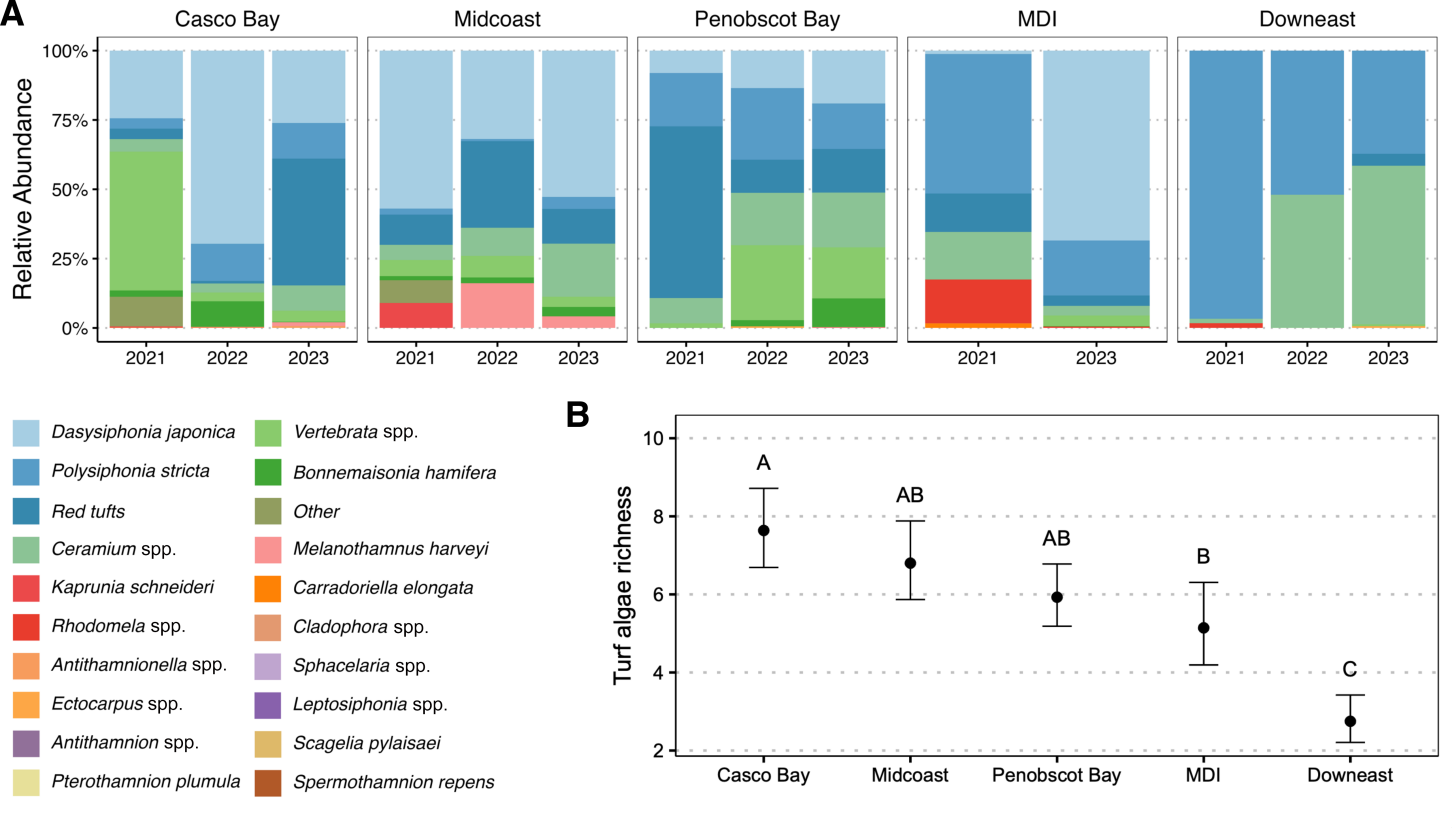
(A) Stacked bars showing the relative abundance of turf algae taxa in each subregion and year (2021–2023). Values are means across all quadrats pooled over sites in that subregion for the given year. Colors denote taxa (species or genus‐level bins). (B) Turf algae richness (mean number of turf taxa per site) by subregion, shown as estimated marginal means ± SE from a Conway–Maxwell–Poisson (COM‐Poisson) mixed model with random intercepts for year and site. Letters indicate Tukey's adjusted pairwise comparisons (*p* < 0.05) (Appendix S1: Table S7).

Our field surveys revealed that, overall, turf algae assemblages were comprised of a diverse consortium of 20 different genera spanning three phyla: Rhodophyta (red algae), Chlorophyta (green algae), and Heterokontophyta (brown algae) (Figure 2a). Yet, the richness and composition of turf communities varied by subregion, with more southerly subregions harboring seven to eight species, intermediate latitudes harboring between five and six species, and the most northerly (Downeast) turf communities consisting of only two to three species (Figure 2b). Notably, turf algae species that were abundant on southern reefs, such as the invasive algae *Dasysiphonia japonica* and *Bonnemaisonia hamifera*, were virtually absent from northern (Downeast) reefs. Instead, native species historically found in low abundance in Maine's kelp forests (e.g., *Ceramium* spp. and *Polysiphonia stricta*) were the main constituents of the low biomass turf algae in the Downeast subregion.

Total macroalgal taxonomic diversity followed an opposite pattern of turf species richness. Kelp forests harbored higher algal species diversity compared to mixed‐state or turf‐dominated reefs. There was a significant, positive, linear relationship between macroalgal diversity (i.e., the Simpson's Index after a Jost transformation) (Appendix S1: Figure S3) and the amount of kelp versus turf algae on the reef (i.e., the kelp‐to‐turf metric). The random effect of site was significant (Appendix S1: Table S12).

### Drivers of turf spread

**FIGURE 3:**
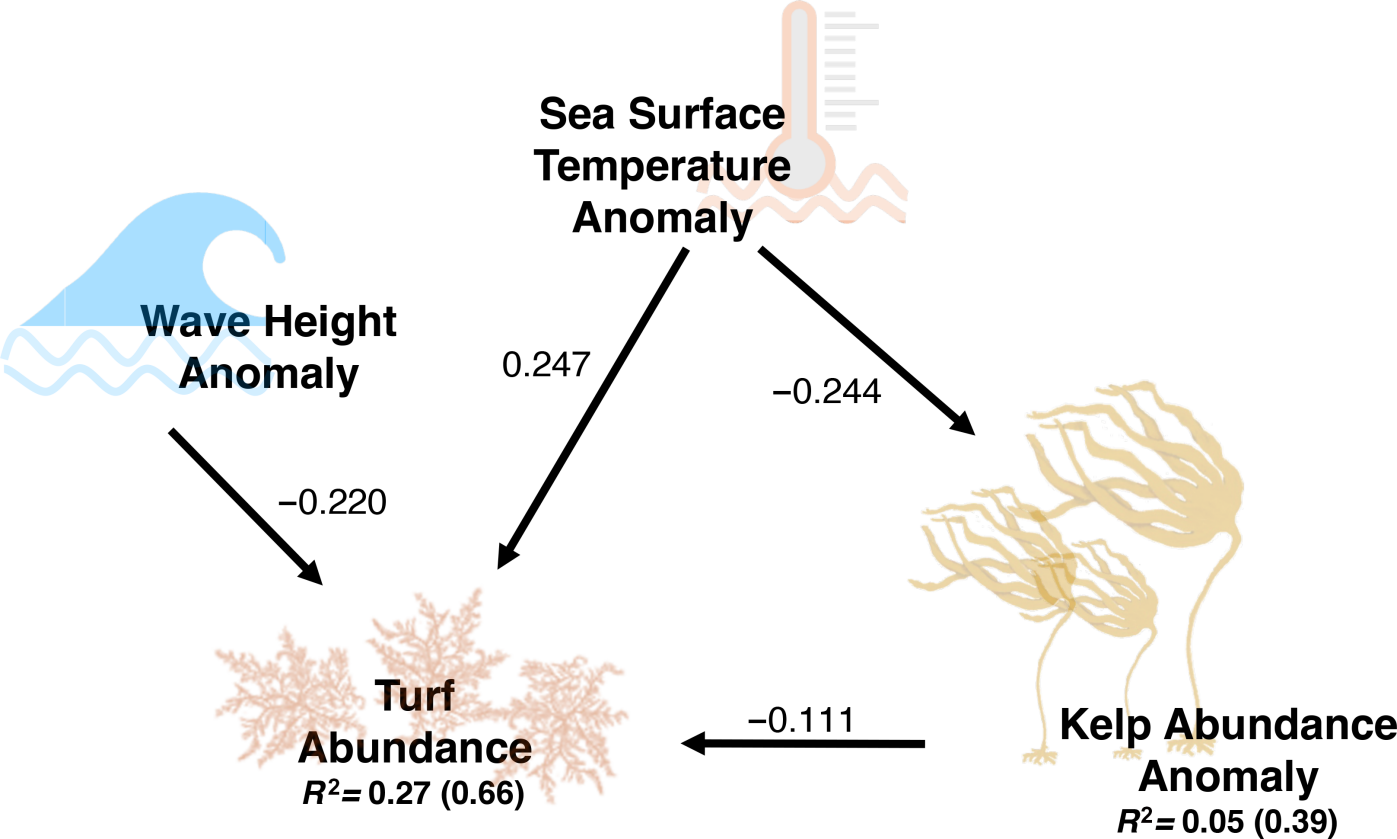
Path diagram from the piecewise structural equation model (SEM). Icons are observed variables and arrows are directed effects. Solid arrows indicate statistically supported paths (*p* < 0.05) (for the full SEM with nonsignificant paths see Appendix S1: Figure S4). Numbers on arrows are standardized path coefficients (effect sizes). “Anomaly” variables are departures from the subregion's long‐term mean (year‐to‐year signal). Negative values denote decreases in the response variable with increasing predictor; positive values denote increases. Values below the endogenous nodes are *R* ^2^ (marginal; conditional). Illustration credits: Dara S. Yiu and Shane P. Farrell.

Our SEM had an adequate fit to the data (Fisher's *C* = 5.18, *p* = 0.269) and revealed both direct and indirect drivers of turf abundance (Figure 3). Specifically, turf abundance was enhanced by local SST anomalies (i.e., deviation from the subregional mean; β_std_ = 0.247, *p* < 0.001) and was reduced by wave height anomalies (β_std_ = −0.220, *p* = 0.024). Turf abundance was also negatively affected by kelp cover anomalies (β_std_ = −0.111, *p* < 0.001), indicating that high kelp cover (relative to the average kelp cover across all 5 years) inhibits turf abundance. Furthermore, kelp cover was negatively impacted by local SST anomalies: as SST increased above the subregional mean, kelp cover declined from its mean (β_std_ = −0.244, *p* = 0.001). Multiplication of the standardized coefficients yielded the indirect effect of SST on turf abundance as mediated by kelp cover (−0.244 × −0.111 = +0.027), which was one‐tenth of the direct effect of SST anomalies on turf abundance (0.247). Thus, we find that SST anomalies promote turf spread via direct effects and, to a lesser extent, indirect effects (via loss of kelp). The subregional means were all nonsignificant but important to include, given the potential for omitted variable bias (Byrnes & Dee, 2025; Suskiewicz et al., 2024) (Appendix S1: Figure S4).

### Consequences of the state shift for seaweed functional diversity

**FIGURE 4:**
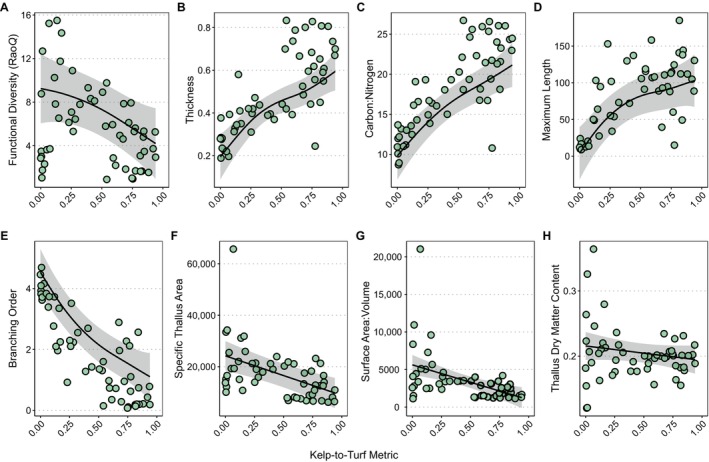
Relationship between kelp‐to‐turf metric (0 = turf‐dominated reef, 1 = kelp‐dominated reef) and (A) community functional diversity (Rao's quadratic entropy) and key algal functional traits, including (B) thickness (mm), (C) carbon‐to‐nitrogen ratio, (D) maximum length (cm), (E) branching order, (F) specific thallus area (mm^2^/g), (G) surface area‐to‐volume ratio (mm^2^/mL), (H) thallus dry matter content. Points (green circles) represent site‐level observations. Black line shows fitted values from a generalized additive model (GAM) holding site and year constant at reference levels. Shaded regions indicate 95% CIs derived from SEs of the predictions (Appendix S1: Tables S12 and S13).

We found that overall seaweed functional diversity (Rao's *Q*) increased with a shift to turf dominance (Figure 4a). However, our community weighted means analysis revealed that specific traits are gained versus lost from the community when reefs experience the state shift (Figure 4b–h). The community on turf‐dominated reefs was characterized by the traits of fine‐scale structural complexity (i.e., more intricate branching) and high surface area (i.e., higher surface area‐to‐volume ratios, and greater specific thallus area). By contrast, the community in kelp forests was dominated by traits of taller canopy heights (i.e., larger thallus size), robustness (i.e., greater thallus thickness), and more nutritious tissues (i.e., higher C:N ratios) (Figure 4b–h; Appendix S1: Table S13). Of note, we also found a linear relationship between turf dominance and functional divergence, and nonlinear relationships between turf dominance and functional richness and functional dispersion, suggesting that trait diversity is maximal during the transition from kelp to turf (Appendix S1: Figure S5).

## DISCUSSION

Ecosystem state shifts are well documented in nature, including coral reefs flipping to algal dominance, lakes shifting from clear to turbid water, or savannas transitioning from grassland to woody dominance (Ling et al., 2015; Scheffer, 2009; Scheffer et al., 2001). Such shifts disrupt ecosystem functioning and ultimately erode key services that support human well‐being (Crépin et al., 2012). Also, these new configurations are often self‐reinforcing, through the emergence of feedback loops (Holling, 1973). Confronting and reversing these shifts thus requires understanding the underlying drivers of change and the processes that prevent recovery (Nyström et al., 2012). However, in the era of climate change, new states are emerging that involve new species and/or new ecological processes (e.g., Ling et al., 2009; Wernberg et al., 2016), making the prediction and management of state shifts increasingly difficult (Hensel et al., 2023). Here, we show that a state shift from kelp forests to turf algae has further progressed across a large marine ecosystem in just a few years (Figure 1) due to the direct and indirect effects of ocean warming (Figure 3), with impacts to the physical architecture and chemical makeup of the biotic habitat (Figure 4). Furthermore, while the stability and reversibility of this novel state shift remain uncertain (Krumhansl et al., 2024), the lasting dominance of turf algae in southern Maine—beyond the generation times of the key species involved (Connell & Sousa, 1983)—and the emergence of ecological feedbacks (Farrell et al., 2025; Feehan et al., 2019) suggest that this shift is stable. Taken together, our findings thus highlight the urgency with which novel state shifts must be studied, monitored, and mitigated to limit their impacts on key ecosystem functions, such as habitat provisioning and carbon storage.

Previously, Suskiewicz et al. (2024) causally linked kelp forest loss along Maine's outer coast (from 2001 to 2018) to unusually high spring and summer seawater temperatures. Through further monitoring of their study sites from 2021 to 2023, we document that the kelp‐to‐turf state shift has since progressed rapidly northward with additional warming (Suskiewicz et al., 2024). Our results further emphasize the critical and swift role that climate change has played in both reducing kelp abundance and facilitating turf proliferation. Just 20 years earlier (2004), turf cover was below 20% in nearly all subregions (Suskiewicz et al., 2024), whereas turf algae now range from 40% to 60% cover on reefs across nearly half of Maine's outer coastline. These findings offer new insight into the ecological dynamics occurring at the warm trailing edge of kelp forest distribution in the Northwest Atlantic (Filbee‐Dexter & Wernberg, 2018; Scheffer & Carpenter, 2003) and show that repeated, fine‐scale monitoring is essential for detecting rapid state shifts under intensifying global change.

Our application of path analysis (SEM) revealed multiple direct and indirect pathways by which climate change is driving turf algae proliferation. First, anomalously warm temperatures both favor turf physiology (a direct effect) and erode the kelp canopy that may otherwise limit turf establishment (an indirect effect) (Bjærke & Rueness, 2004; Breeman et al., 1988), together enhancing the spread of turf algae on Maine's coast (Figure 3). Second, reductions in turf cover with increasing wave energy (relative to the subregional mean) suggest that disturbance‐driven detachment of turf algae outweighs any potential gains that turf algae incur from fragment‐mediated dispersal in this system, aligning with previous work showing that physical disturbances suppress understory algae (Cecere et al., 2011; Duggins et al., 1990; Husa & Sjøtun, 2006; Irving & Connell, 2006; Kennelly, 1987; Mulders et al., 2022; Santelices & Ojeda, 1984; Smale et al., 2020; Toohey, 2007). Although turf establishment and persistence can also depend on top‐down forcing (e.g., elevated levels of herbivory that open space for turf and prevent kelp re‐establishment, as in Tasmania; Ruiz‐Ruiz et al., 2025), sea urchin herbivores were so scarce during our study that we could not include them in the SEM. Together, these findings reiterate the idea that ocean warming is now governing the outcomes of top‐down forcing in this ecosystem (Suskiewicz et al., 2024).

In our study, the long‐term subregional means (SST, waves, kelp) were nonsignificant once local anomalies for these predictors were included in the SEM, indicating that the year‐to‐year deviation from subregional baselines—not the absolute difference between subregions—is the signal most predictive of turf change. These findings align with the results of Suskiewicz et al. (2024), who found that unusually warm spring/summer SST anomalies drove kelp declines along the Maine outer coast from 2001 to 2018, and who in turn argued that the thermal “tipping point” for the kelp‐to‐turf transition likely differs among subregions—because of differing oceanographic conditions, limited kelp dispersal and population connectivity (Breton et al., 2018), and resultant kelp adaptations to local thermal histories. Because subregional patterns of kelp and turf algae abundance track SST anomaly magnitude/frequency more than absolute conditions (Figure 3; Suskiewicz et al., 2024), such dynamics and local specificity must be incorporated into modeling and experimental studies that aim to predict the future pace and spread of this state shift, and inform the management or recovery of regional kelp forests.

While several studies have documented the shift from kelp‐to‐turf dominance on temperate reefs (Dijkstra et al., 2017; Feehan et al., 2019; Suskiewicz et al., 2024; Wernberg et al., 2013), few have resolved the species composition of the resultant turf algae community. We show that these communities are diverse and include both native and non‐native taxa, which vary in species richness and community composition across subregions (Figure 2). Notably, the invasive *D. japonica* was the dominant species in southern subregions (Figure 2). The dominance of *D. japonica* is likely due to its broad thermal tolerance (0–30°C) and optimal growth around 19–25°C, conditions which closely match contemporary summer temperatures in the southern Gulf of Maine. *D. japonica*'s physiology, coupled with our SEM results indicating strong positive effects of warming sea surface temperatures on turf, underscores the role of species introductions in amplifying ecosystem state shifts. Our study is therefore one of many examples (documented in forests, grasslands, wetlands, aquatic, and riparian ecosystems) showing how climate change is facilitating species introductions, with resultant impacts on ecosystem function and stability (Crowl et al., 2008; Gaertner et al., 2014; Johnstone et al., 2016; Sorte et al., 2010; Strange et al., 2018; Yiu et al., 2025). Likewise, this study indicates that species‐level monitoring of state shifts is critical, given that individual turf and kelp species have distinct functional traits (i.e., chemical, morphological, and physiological attributes; Figure 4) that each impact ecosystem functioning, resilience, and state shift dynamics in unique and specific ways (Dijkstra et al., 2017; Farrell et al., 2025).

Using our high‐resolution biomass data, we examined how increasing turf cover influences algal community taxonomic and functional diversity. Our results reveal a decoupling between taxonomic and functional diversity along the kelp–turf continuum (Figure 4; Appendix S1: Figure S3). Kelp forests had greater taxonomic diversity yet lower functional diversity than turf‐dominated reefs, likely due to the kelp canopy imposing strong environmental filtering on the substrate below (e.g., shade and whiplash), favoring a canopy‐tolerant trait set. Species diversity can increase without expanding trait space with strong environmental forces or competitively dominant species generating functional redundancy. Similar patterns were found in mammals where functional diversity plateaued with increasing species richness, or in alpine grasslands where functional diversity increased when species diversity decreased (Niu et al., 2015; Oliveira et al., 2016).

Comparing kelp forests to turf reefs, we observed significant declines in algal traits associated with investment in large, three‐dimensional structures typical of foundation species, such as maximum length, thickness, and C:N ratios, indicating reduced allocation to carbon‐rich, durable tissues at the community level. These traits also underpin the ecosystem functions (e.g., habitat provisioning, carbon‐based food production) expected of foundation species where competition for light and mechanical stress favor robust structural investment (Mauffrey et al., 2020). Community‐level trait contractions along environmental stress gradients are also found in intertidal seaweed assemblages. In these systems, as stress increases, functional redundancy is eroded, with varied responses to trait diversity (Cappelatti et al., 2020).

On turf‐dominated reefs, the algal community exhibited traits optimized for rapid growth and resource acquisition, such as higher branching order, specific thallus area, and surface area‐to‐volume ratios, which are known to enhance nutrient uptake and photosynthetic efficiency. Thus, as kelp forests collapse, the ecosystem loses large, three‐dimensional habitats, disrupting predator–prey dynamics (Dijkstra et al., 2019; O'Brien et al., 2018; Pessarrodona et al., 2021). Further, the loss of kelp‐driven primary production can reduce carbon production potential and the flow of fixed carbon into coastal food webs (Pessarrodona, Assis, et al., 2022; Pessarrodona, Vergés, et al., 2022; Ramsay‐Newton et al., 2017). Meanwhile, turf reefs, through their highly branched structures, increase small interstitial spaces that benefit meso‐invertebrates like amphipods and isopods (Dijkstra et al., 2017) and overall appear tuned for rapid dispersal and turnover. While individual empirical measures of ecosystem functioning are essential for understanding the consequences of state shifts (e.g., Pessarrodona et al., 2021; Yiu et al., 2025), our functional trait analysis suggests a net decline of functioning with the loss of foundational kelps. Moreover, our trait analysis provides further context needed to investigate the food web consequences of this novel state shift.

Lastly, our findings underscore the urgency of curbing global carbon emissions to halt further expansion of this state shift, and the need to develop regional management approaches for combating its impacts. Likewise, adaptive management to safeguard remaining kelp forests—which still span over a thousand kilometers of continuous coastline in Maine—remains essential. Efforts to mitigate co‐stressors and develop and implement restoration strategies, such as augmenting kelp abundance and removing turf algae from the “front lines” of this state shift, may help sustain ecosystem functions and services, buying valuable time while global emissions are reduced (Wood et al., 2021). Once ocean warming is curbed, this state shift may be reversible (Krumhansl et al., 2024) via natural recovery processes or active restoration efforts. High‐resolution mechanistic studies such as ours, carried out in a “microcosm” of global change, can inform such efforts in this and other imperiled ecosystems.

## AUTHOR CONTRIBUTIONS

Shane P. Farrell and Douglas B. Rasher conceptualized the study. Shane P. Farrell, Dara S. Yiu, and Douglas B. Rasher designed the study. All authors collected the data. Shane P. Farrell and Jonathan S. Lefcheck analyzed the data. Shane P. Farrell and Douglas B. Rasher wrote the manuscript. All authors edited the final version of the manuscript.

## FUNDING INFORMATION

This project was supported through an United States National Science Foundation EPSCoR (OIA‐1489227) given to the University of Maine System from the EPSCoR RII Track‐1 program (DBR) and The Louise H. & David S. Ingalls Foundation (DBR).

## CONFLICT OF INTEREST STATEMENT

The authors declare no conflicts of interest.

## Supporting information


Appendix S1.

## Data Availability

Data and code (Farrell et al., 2026) are available in Dryad at https://doi.org/10.5061/dryad.rr4xgxdk2.
