# Supplementary material for: From kelp forests to turf reefs: Patterns, drivers, and impacts to functional diversity
Source: Ecology. 2026 May 17;107:e70408. doi: 10.1002/ecy.70408 (PMC13180503; doi:10.1002/ecy.70408)
Supplement: Supplementary file 1 — Appendix S1. [file ECY-107-e70408-s001.pdf]

Appendix S1

From kelp forests to turf reefs: Patterns, drivers, and impacts to functional diversity

Shane P. Farrell, Dara S. Yiu, Stuart K. Ryan, Rene D. Francolini, Courtney E. Stuart,

Jonathan S. Lefcheck, Yasmina M. Shah Esmaeili, and Douglas B. Rasher

Ecology

Table S1. Summary of site locations and visits in 2018, 2021, 2022, and 2023. Shaded squares represent whether a site was visited in a given year.

Site	Sub-region	Latitude	Longitude	2018	2021	2022	2023
Cape Elizabeth	Casco	43.5632	-70.1968	■	■	■	■
Jewell Island	Casco	43.6866	-70.0843	■			■
Halfway Rock	Casco	43.6546	-70.0373	■	■	■	■
Bold Dick Rock	Casco	43.7131	-69.9409	■			■
Cape Small	Casco	43.7012	-69.8399	■	■	■	■
Seguin Island	Midcoast	43.7051	-69.7541	■			■
Damariscove Island	Midcoast	43.7565	-69.6188	■	■	■	■
Thrumcap Island	Midcoast	43.8235	-69.5505				■
Pemaquid Point	Midcoast	43.8389	-69.5019	■	■		■
Monhegan Island	Midcoast	43.7679	-69.3224	■			
Allen Island	Midcoast	43.8614	-69.3237	■	■	■	■
Metinic Island	Penobscot	43.8679	-69.1288	■	■	■	■
Andrews Island	Penobscot	43.9959	-69.0515	■	■		■
Hurricane Island	Penobscot	44.0304	-68.8893	■	■		■
Merchant Island	Penobscot	44.1057	-68.6535	■			■
Greens Island	Penobscot	44.0246	-68.8612	■			
Isle au Haut	Penobscot	44.0149	-68.6078	■	■	■	■
Kimball Island	Penobscot	44.0731	-68.6708	■			
Marshall Island	Penobscot	44.1011	-68.5088		■	■	■
Long Island	MDI	44.1026	-68.3438	■			■
Little Duck Island	MDI	44.1709	-68.2420	■	■		■
Little Moose Island	MDI	44.3345	-68.0525	■			
Schoodic Island	MDI	44.3254	-68.0304		■		■
Western Island	MDI	44.389	-67.9672				■
Dyer Point	MDI	44.4110	-67.9307				■
Petit Manan Island	Downeast	44.3641	-67.8685		■		■
Steele Harbor Island	Downeast	44.4822	-67.5276	■			
Flint Island	Downeast	44.4718	-67.7859	■			■
Little Drisko Island	Downeast	44.4823	-67.6669		■	■	■
Crumple Island	Downeast	44.4447	-67.5976	■		■	■
Great Spruce Island	Downeast	44.5562	-67.4914	■			■
Ram Island	Downeast	44.5739	-67.3962	■	■	■	■

Sites/Sub-region	2018	2021	2022	2023
Casco Bay	5	3	3	5
Midcoast	5	2	2	5
Penobscot Bay	7	6	3	6
MDI	3	2	0	5
Downeast	5	3	3	6
Total sites	25	16	11	27

Table S2: Summary of the kelp model output for a generalized linear mixed model (GLMM) assessing temporal changes in kelp cover across sub-regions of the Maine coast. The table reports key model fit metrics (including AIC, BIC, log-likelihood) along with parameter estimates, standard errors, z-values with high and low confidence intervals, and *p* values for each effect. Year effects are compared to the baseline year (2018) to assess significant changes in kelp cover between 2018 and each subsequent year. The models included a conditional, zero-inflation, and dispersion component.

**Observations**	**Random_groups**	**AIC**	**BIC**	**LogLik**	**Convergence**
632	Site=32; Obs=632	-354.5	-230	205.3	Model converged without warnings

**Component**	**Term**	**Estimate**	**Std Error**	**Statistic**	**CI Low**	**CI High**	**P Value**
Conditional	(Intercept)	-2.59	0.295	-8.77	-3.168	-2.011	<0.001
Conditional	Year_c	-0.041	0.063	-0.65	-0.165	0.083	0.517
Conditional	RegionDowneast	2.131	0.355	6	1.435	2.827	<0.001
Conditional	RegionMDI	2.833	0.357	7.94	2.134	3.532	<0.001
Conditional	RegionMidcoast	1.287	0.364	3.53	0.573	2.001	<0.001
Conditional	RegionPenobscot Bay	1.661	0.344	4.83	0.987	2.336	<0.001
Conditional	Year_c:RegionDowneast	0.03	0.069	0.43	-0.105	0.165	0.665
Conditional	Year_c:RegionMDI	-0.113	0.076	-1.49	-0.263	0.036	0.137
Conditional	Year_c:RegionMidcoast	-0.207	0.076	-2.74	-0.356	-0.059	0.006
Conditional	Year_c:RegionPenobscot Bay	-0.074	0.072	-1.02	-0.215	0.068	0.308
Zero-Inflated	(Intercept)	-0.109	0.255	-0.43	-0.609	0.391	0.669
Zero-Inflated	Year_c	0.127	0.064	1.98	0.001	0.252	0.048
Zero-Inflated	RegionDowneast	-23.684	10446.776	0	-20498.989	20451.621	0.998
Zero-Inflated	RegionMDI	-23.397	11119.239	0	-21816.704	21769.91	0.998
Zero-Inflated	RegionMidcoast	-2.072	0.319	-6.49	-2.698	-1.446	<0.001
Zero-Inflated	RegionPenobscot Bay	-2.512	0.32	-7.86	-3.139	-1.886	<0.001
Dispersion	(Intercept)	2.89	0.367	7.88	2.171	3.609	<0.001
Dispersion	Year_c	-0.106	0.1	-1.05	-0.302	0.091	0.292
Dispersion	RegionDowneast	-0.092	0.465	-0.2	-1.004	0.82	0.843
Dispersion	RegionMDI	0.51	0.51	1	-0.49	1.51	0.317
Dispersion	RegionMidcoast	-0.542	0.427	-1.27	-1.378	0.295	0.204
Dispersion	RegionPenobscot Bay	-0.577	0.411	-1.4	-1.383	0.228	0.16
Dispersion	Year_c:RegionDowneast	0.038	0.128	0.3	-0.212	0.289	0.764
Dispersion	Year_c:RegionMDI	-0.258	0.138	-1.87	-0.529	0.012	0.061
Dispersion	Year_c:RegionMidcoast	0.153	0.124	1.24	-0.089	0.395	0.216
Dispersion	Year_c:RegionPenobscot Bay	0.012	0.119	0.1	-0.221	0.244	0.921

Table S3: Summary of the turf model output for a generalized linear mixed model (GLMM) assessing temporal changes in turf cover across sub-regions of the Maine coast. The table reports key model fit metrics (including AIC, BIC, log-likelihood) along with parameter estimates, standard errors, z-values with high and low confidence intervals, and *p* values for each effect. Year effects are compared to the baseline year (2018) to assess significant changes in turf cover between 2018 and each subsequent year. The models included a conditional, zero-inflation, and dispersion component.

Observations	Random_groups	AIC	BIC	logLik	Convergence
632	Site=32; Obs=632	-825.3	-718.5	436.6	Model converged without warnings

Component	Term	Estimate	Std Error	Statistic	CI Low	CI High	P Value
Conditional	(Intercept)	-1.766	0.236	-7.49	-2.228	-1.304	<0.001
Conditional	Year_c	0.218	0.036	6.01	0.147	0.289	<0.001
Conditional	RegionDowneast	-0.834	0.341	-2.45	-1.502	-0.166	0.014
Conditional	RegionMDI	-1.06	0.344	-3.08	-1.735	-0.386	0.002
Conditional	RegionMidcoast	0.578	0.326	1.77	-0.062	1.218	0.077
Conditional	RegionPenobscot Bay	-0.581	0.305	-1.9	-1.178	0.017	0.057
Conditional	Year_c:RegionDowneast	-0.16	0.057	-2.83	-0.272	-0.049	0.005
Conditional	Year_c:RegionMDI	0.13	0.06	2.15	0.011	0.248	0.032
Conditional	Year_c:RegionMidcoast	-0.04	0.055	-0.74	-0.147	0.067	0.462
Conditional	Year_c:RegionPenobscot Bay	0.033	0.05	0.66	-0.065	0.131	0.508
Zero-Inflated	(Intercept)	-2.277	0.478	-4.76	-3.215	-1.34	<0.001
Zero-Inflated	Year_c	-0.483	0.109	-4.44	-0.696	-0.27	<0.001
Zero-Inflated	RegionDowneast	0.814	0.589	1.38	-0.341	1.968	0.167
Zero-Inflated	RegionMDI	-0.23	0.756	-0.3	-1.713	1.252	0.761
Zero-Inflated	RegionMidcoast	-0.214	0.695	-0.31	-1.575	1.147	0.758
Zero-Inflated	RegionPenobscot Bay	0.155	0.594	0.26	-1.009	1.319	0.794
Dispersion	(Intercept)	2.295	0.162	14.2	1.979	2.612	<0.001
Dispersion	Year_c	-0.105	0.036	-2.95	-0.175	-0.035	0.003
Dispersion	RegionDowneast	0.427	0.189	2.26	0.057	0.797	0.024
Dispersion	RegionMDI	0.267	0.203	1.31	-0.131	0.665	0.189
Dispersion	RegionMidcoast	-0.417	0.177	-2.36	-0.764	-0.07	0.018
Dispersion	RegionPenobscot Bay	0.194	0.172	1.13	-0.143	0.532	0.259

Table S4. Estimated marginal trends for change in kelp cover by sub-region. For each sub-region, the table reports the slope on the logit link (change per year), its standard error (SE)**,** Wald z-ratio, and 95% CI on the logit scale (CI Logit). Slopes are back-transformed to odds ratios (OR) describing the multiplicative change in the odds of kelp cover per year, with 95% CI (CI OR), and to percent change per year (Pct Change) with 95% CI (CI Pct). *p* values test the null hypothesis of no temporal trend (slope = 0). Trends are estimated marginal trends from the fitted model (year × sub-region fixed effects), evaluated with predictors at their reference levels and random effects at their population means. Kelp percent cover was scaled and bounded to (0,1) prior to modeling.

Sub-region	Slope Logit	SE	Z Ratio	CI Logit	OR	CI OR	Pct Change	CI Pct	P Value
Casco Bay	-0.041	0.063	-0.65	(-0.165, 0.083)	0.96	(0.848, 1.087)	-4	(-15.2, 8.7)	0.517
Downeast	-0.011	0.027	-0.41	(-0.064, 0.042)	0.989	(0.938, 1.043)	-1.1	(-6.2, 4.3)	0.683
MDI	-0.154	0.042	-3.64	(-0.237, -0.071)	0.857	(0.789, 0.931)	-14.3	(-21.1, -6.9)	<0.001
Midcoast	-0.248	0.041	-5.99	(-0.33, -0.167)	0.78	(0.719, 0.846)	-22	(-28.1, -15.4)	<0.001
Penobscot Bay	-0.115	0.035	-3.3	(-0.183, -0.046)	0.892	(0.833, 0.955)	-10.8	(-16.7, -4.5)	<0.001

Table S5. Estimated marginal trends for changes in turf cover by sub-region. For each sub-region, the table reports the slope on the logit link (change per year), its standard error (SE)**,** Wald z-ratio, and 95% CI on the logit scale (CI Logit). Slopes are back-transformed to odds ratios (OR) describing the multiplicative change in the odds of kelp cover per year, with 95% CI (CI OR), and to percent change per year (Pct Change) with 95% CI (CI Pct). *p* values test the null hypothesis of no temporal trend (slope = 0). Trends are estimated marginal trends from the fitted model (year × sub-region fixed effects), evaluated with predictors at their reference levels and random effects at their population means. Turf percent cover was scaled and bounded to (0,1) prior to modeling.

Sub-region	Slope Logit	SE	Z Ratio	CI Logit	OR	CI OR	Pct Change	CI Pct	P Value
Casco Bay	0.218	0.036	6.01	(0.147, 0.289)	1.243	(1.158, 1.334)	24.3	(15.8, 33.4)	<0.001
Downeast	0.057	0.046	1.25	(-0.032, 0.147)	1.059	(0.968, 1.158)	5.9	(-3.2, 15.8)	0.21
MDI	0.347	0.05	6.9	(0.249, 0.446)	1.415	(1.282, 1.562)	41.5	(28.2, 56.2)	<0.001
Midcoast	0.178	0.042	4.21	(0.095, 0.26)	1.194	(1.099, 1.297)	19.4	(9.9, 29.7)	<0.001
Penobscot Bay	0.251	0.035	7.07	(0.181, 0.32)	1.285	(1.199, 1.377)	28.5	(19.9, 37.7)	<0.001

Table S6. Summary of the generalized linear mixed model (GLMM) evaluating sub-regional differences in turf community richness. The table reports sample size (Observations)**,** random-effects structure**,** AIC/BIC**,** log-likelihood, and convergence diagnostics, followed by fixed-effect coefficients for sub-region. Coefficients are shown as estimates on the log scale with standard errors (SE), Wald z statistics, 95% confidence intervals on the log scale (CI log), and *p* values. The Intercept corresponds to the model reference sub-region (Midcoast); other sub-region coefficients represent contrasts relative to the reference**.** Negative estimates (and odds < 1 if exponentiated) indicate lower richness than the reference.

**Observations**	**Random groups**	**AIC**	**BIC**	**logLik**	**Convergence**
54	Year=3; Site=27	208	223.9	-96	Model converged without warnings

**Term**	**Estimate Log**	**SE**	**z**	**CI Log**	**P Value**
(Intercept)	1.917	0.075	25.5	(1.77, 2.064)	<0.001
RegionPenobscot Bay	-0.137	0.102	-1.35	(-0.336, 0.062)	0.177
RegionCasco Bay	0.116	0.101	1.15	(-0.082, 0.314)	0.251
RegionDowneast	-0.905	0.135	-6.72	(-1.169, -0.641)	<0.001
RegionMDI	-0.279	0.128	-2.17	(-0.531, -0.028)	0.03

Table S7. Pairwise sub-regional contrasts in turf richness from the GLMM. The table reports ratios of estimated marginal means on the response scale; a ratio > 1 indicates higher mean richness in the first sub-region listed (numerator) relative to the second (denominator), while a ratio < 1 indicates lower biomass. SE is the standard error of the z_ratio, the Wald test statistic, and *p* values are Tukey-adjusted for multiple comparisons across all regional pairs.

Contrast	Ratio	CI Low	CI High	SE	Z Ratio	P Value
Midcoast / Penobscot Bay	1.147	0.869	1.513	0.116	1.35	0.66
Midcoast / Casco Bay	0.89	0.676	1.173	0.09	-1.148	0.781
Midcoast / Downeast	2.473	1.712	3.571	0.333	6.718	<0.001
Midcoast / MDI	1.322	0.931	1.877	0.17	2.175	0.189
Penobscot Bay / Casco Bay	0.776	0.597	1.009	0.075	-2.637	0.064
Penobscot Bay / Downeast	2.156	1.508	3.082	0.282	5.863	<0.001
Penobscot Bay / MDI	1.153	0.821	1.619	0.144	1.142	0.784
Casco Bay / Downeast	2.777	1.945	3.965	0.363	7.82	<0.001
Casco Bay / MDI	1.485	1.059	2.083	0.184	3.186	0.013
Downeast / MDI	0.535	0.352	0.811	0.082	-4.097	<0.001

Table S8. SEM sub-model 1, a linear mixed-effects model predicting log-transformed turf cover from group mean-centered SST, kelp cover, and wave height and sub-regional mean covariates. The model was fit by REML with random intercepts of year nested within site within sub-region and a continuous CAR(1) correlation within year/site/sub-region. The table reports unstandardized coefficients (β), standard errors (SE), denominator degrees of freedom (DF), t values, and *p* values.

logLik	AIC	BIC	Observations	Regions	Sites	Years within Sites
-316.3813	656.7626	709.7044	616	5	30	77

Term	Estimate (β)	CI Low	CI High	DF	t Value	P Value
(Intercept)	-6.868	-13.009	-0.727	538	-2.197	0.028
kelp_centered_reg	-0.002	-0.004	-0.001	538	-3.406	0.001
Sub-region_mean_kelp	0.011	0.157	0.575	1	1.546	0.365
mean_sst_centered_reg	0.366	-0.081	0.104	45	3.529	0.001
Sub-region_mean_sst	0.796	-2.968	4.560	1	2.688	0.227
wave_1yr_centered_reg	-0.542	-5.414	5.389	45	-2.325	0.025
Sub-region_mean_wave	-0.012	-1.011	-0.072	1	-0.029	0.982

Table S9. SEM sub-model 2, a linear mixed-effects model predicting kelp cover from group mean-centered SST and sub-regional mean covariates. The model was fit by REML with random intercepts of year nested within site within sub-region and a continuous CAR(1) correlation within year/site/sub-region. The table reports unstandardized coefficients (β), standard errors (SE), denominator degrees of freedom (DF), t values, and *p* values.

logLik	AIC	BIC	Observations	Sites	Years within Sites
-2771.627	5559.255	5594.588	616	30	77

Term	Estimate (β)	CI Low	CI High	DF	t Value	P Value
(Intercept)	18.252276	-249.71	286.2149478	539	0.133803	0.893
mean_sst_centered_reg	-16.488714	-26.1819	-6.7955104	46	-3.424055	0.001
Sub-region_mean_sst	-1.434765	-27.9871	25.1175219	27	-0.110872	0.912
Sub-region_mean_kelp	-0.000806	-0.65616	0.6545493	27	-0.002522	0.998

Table S10. Piecewise structural equation model (SEM) summarizing links between turf cover and environmental covariates (sub-regional and group mean-centered kelp cover, SST and waves). Sub-models were fit as linear mixed-effects models with random intercepts (sub-region/site/year) and a continuous CAR(1) temporal correlation estimated by REML. Table reports global goodness-of-fit (Fisher's C and Chi-square) with degrees of freedom (df) and *p* values with marginal and conditional R^2 for each endogenous response. Tests of directed separation are presented with degrees of freedom, test statistic and *p* value. Finally, path coefficients for all directed edges: unstandardized estimates (β), standard errors (SE), denominator degrees of freedom (df), test statistic (t), *p* values, standardized coefficients (Std. β) and confidence intervals (CI).

Goodness of fit		
**Metric**	**DF**	**P Value**
Chi-Squared	2	16.682
Fisher's C	4	5.186

R²		
**Response**	**Marginal R²**	**Conditional R²**
log_turf	0.27	0.66
kelp_centered_reg	0.06	0.4

Tests of directed separation			
**Independence claim**	**DF**	**Test statistic**	**P Value**
kelp_centered_reg ~ wave_1yr_centered_reg + …	45	−1.623	0.111
kelp_centered_reg ~ region_mean_wave + …	26	0.43	0.67

Path Coefficients									
**Response**	**Predictor**	**Estimate (β)**	**SE**	**DF**	**Test statistic**	**P Value**	**Std. β**	**CI Low**	**CI High**
log_turf	kelp_centered_reg	−0.002	0.0007	538	−3.406	0.0007	−0.111	-0.004	-0.001
log_turf	Sub-region_mean_kelp	0.011	0.007	1	1.546	0.3654	0.503	-0.081	0.104
log_turf	mean_sst_centered_reg	0.365	0.103	45	3.529	0.001	0.247	0.157	0.575
log_turf	Sub-region_mean_sst	0.796	0.296	1	2.688	0.2267	0.885	-2.968	4.560
log_turf	wave_1yr_centered_reg	−0.541	0.232	45	−2.325	0.0246	−0.219	-1.011	-0.072
log_turf	Sub-region_mean_wave	−0.012	0.425	1	−0.028	0.9818	−0.002	-5.414	5.389
kelp_centered_reg	mean_sst_centered_reg	−16.488	4.815	46	−3.424	0.0013	−0.243	-26.182	-6.796
kelp_centered_reg	Sub-region_mean_sst	−1.434	12.940	27	−0.110	0.9125	−0.034	-27.987	25.118
kelp_centered_reg	Sub-region_mean_kelp	−0.0008	0.319	27	−0.002	0.998	−0.000	-0.656	0.655

Table S11. A list of the seaweed species we collected (i.e., those not in Mauffrey et al. 2020) and associated site(s) of collection. Many of these species are found in extremely low abundance in nature and thus collection may have occurred at only one site.

Species	Plant replicates	Sites Collected	Traits Measured
Agarum clathratum	2	Metinic Island	All
Ahnfeltia spp	2	Little Drisko Island	All
Bonnemaisonia hamifera	4	Metinic Island	All
Codium fragile	2	Metinic Island, Damariscove Island	All
Dasysiphonia japonica	4	Metinic Island, Damriscove Island	All
Desmarestia viridis	3	Petit Manan Island, Ram Island	All
Euthora cristata	3	Ram Island	All
Melanothamnus harveyi	4	Allen Island	All
Membranoptera fabriciana	2	Ram Island	All
Phycodrys fimbriata	2	Great Spruce Island, Ram Island	All
Phyllophora pseudoceranoides	3	Metinic Island	All
Palmaria palmata	1	Metinic Island	All
Polysiphonia stricta	4	Great Spruce Island, Ram Island	All
Polyides rotunda	1	Crumple Island	All
Ptilota serrata	2	Ram Island	All
Red tubes	4	Great Spruce Island, Ram Island	All

Table S12. Summary of generalized additive model (GAM) outputs assessing the relationships between the kelp-to-turf metric and species diversity and functional diversity metrics, including Simpson's Diversity Index, functional richness, functional dispersion, functional evenness, Rao's quadratic entropy (Rao's *Q*), and functional divergence. Each model reports the explained deviance, R^2 values, and REML score, as well as significance tests for smooth terms.

Model	R^2	Deviance Explained	REML	
Simpson's Diversity	0.4	54%	63.945	
**Term**	**edf**	**Ref.df**	**F**	**P Value**
s(Kelp-to-turf metric)	1	1	7.928	0.007
s(Year)	0.132	2	0.073	0.344
s(Site)	10.971	26	0.831	0.028

Model	R^2	Deviance Explained	REML	
Rao *Q*	0.56	70%	137.88	
**Term**	**edf**	**Ref.df**	**F**	**P Value**
s(Kelp-to-turf metric)	1.53	1.793	7.227	0.011
s(Year)	0	2	0	0.623
s(Site)	15.194	26	1.713	0.002

Model	R^2	Deviance Explained	REML	
Functional Richness	0.64	77%	40.64	
**Term**	**edf**	**Ref.df**	**F**	**P Value**
s(Kelp-to-turf metric)	2.345	2.781	3.635	0.021
s(Year)	0	2	0	0.48
s(Site)	16.614	26	2.351	<0.001

Model	R^2	Deviance Explained	REML	
Functional Dispersion	0.55	69%	63.928	
**Term**	**edf**	**Ref.df**	**F**	**P Value**
s(Kelp-to-turf metric)	1.963	2.329	4.307	0.019
s(Year)	0	2	0	0.618
s(Site)	14.973	26	1.636	0.002

Model	R^2	Deviance Explained	REML	
Functional Evenness	0.12	19%	-28.355	
**Term**	**edf**	**Ref.df**	**F**	**P Value**
s(Kelp-to-turf metric)	2.815	3.473	1.802	0.156
s(Year)	0	2	0	0.713
s(Site)	1.3	26	0.053	0.396

Model	R^2	Deviance Explained	REML	
Functional Divergence	0.64	76%	-25.935	
**Term**	**edf**	**Ref.df**	**F**	**P Value**
s(Kelp-to-turf metric)	1	1	19.307	<0.001
s(Year)	1.113	2	1.466	0.133
s(Site)	14.713	26	1.69	0.002

Table S13. Summary of generalized additive model (GAM) outputs evaluating the relationships between the kelp-to-turf metric and key algal functional traits, including thickness, carbon-to-nitrogen ratio, maximum length, branching order, thallus dry matter content, surface area-to-volume ratio, and surface thallus area. Each model reports the explained deviance, R^2 values, and REML scores, along with significance tests for smooth terms

**Model**	**R^2**	**Deviance Explained**	**REML**	
Branching Order	0.86	91%	58.254	
**Term**	**edf**	**Ref.df**	**F**	**P Value**
s(Kelp_turf_pct_metric)	2.374	2.804	37.242	<0.001
s(Year)	1.121	2	1.62	0.109
s(Site)	14.488	26	1.699	0.001

**Model**	**R^2**	**Deviance Explained**	**REML**	
Thallus Dry Matter Content	0.03	6%	-89.192	
**Term**	**edf**	**Ref.df**	**F**	**P Value**
s(Kelp_turf_pct_metric)	1	1	1.673	0.202
s(Year)	0.679	2	0.477	0.253
s(Site)	0	26	0	0.594

**Model**	**R^2**	**Deviance Explained**	**REML**	
Thickness	0.94	97%	-45.368	
**Term**	**edf**	**Ref.df**	**F**	**P Value**
s(Kelp_turf_pct_metric)	2.702	3.269	14.471	<0.001
s(Year)	0	2	0	0.765
s(Site)	23.274	26	11	<0.001

**Model**	**R^2**	**Deviance Explained**	**REML**	
Surface Area:Volume	0.2	22%	491.31	
**Term**	**edf**	**Ref.df**	**F**	**P Value**
s(Kelp_turf_pct_metric)	1	1	14.608	<0.001
s(Year)	0	2	0	0.651
s(Site)	0.002	26	0	0.627

**Model**	**R^2**	**Deviance Explained**	**REML**	
Specific Thallus Area	0.29	34%	549.45	
**Term**	**edf**	**Ref.df**	**F**	**P Value**
s(Kelp_turf_pct_metric)	1	1.001	15.679	<0.001
s(Year)	0	2	0	0.628
s(Site)	3.261	26	0.151	0.289

**Model**	**R^2**	**Deviance Explained**	**REML**	
Carbon:Nitrogen	0.9	94%	128.96	
**Term**	**edf**	**Ref.df**	**F**	**P Value**
s(Kelp_turf_pct_metric)	2.09	2.507	20.524	<0.001
s(Year)	0	2	0	0.67
s(Site)	21.19	26	4.912	<0.001

**Model**	**R^2**	**Deviance Explained**	**REML**	
Maximum Length	0.77	86%	256.82	
**Term**	**edf**	**Ref.df**	**F**	**P Value**
s(Kelp_turf_pct_metric)	2.532	2.986	12.967	<0.001
s(Year)	1.111	2	1.433	0.153
s(Site)	16.369	26	1.949	0.001

Figure S1. Estimated annual change in kelp percent cover by sub-region from a zero-inflated beta mixed model. Points are *emmeans*-derived slopes (percent change per year) for each sub-region, and vertical bars are 95% Wald confidence intervals computed from the year x sub-region interaction on the model scale and back-transformed to the response scale. The dashed line denotes no change per year.

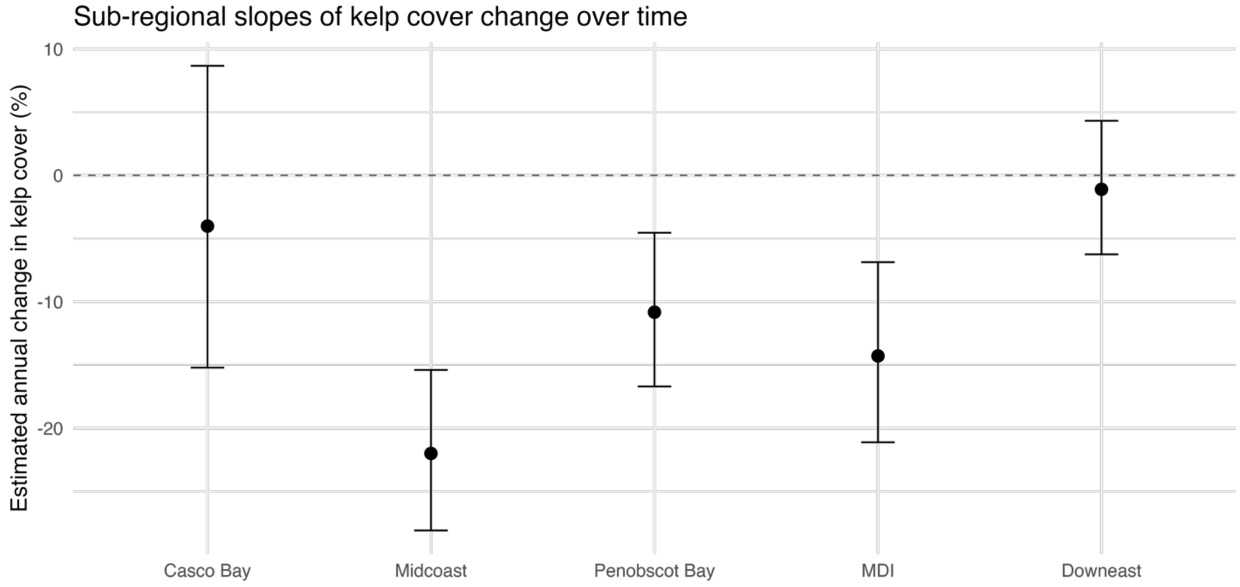

Figure S2. Estimated annual change in turf percent cover by sub-region from a zero-inflated beta mixed model. Points are *emmeans*-derived slopes (percent change per year) for each sub-region, and vertical bars are 95% Wald confidence intervals computed from the year x sub-region interaction on the model scale and back-transformed to the response scale. The dashed line denotes no change per year.

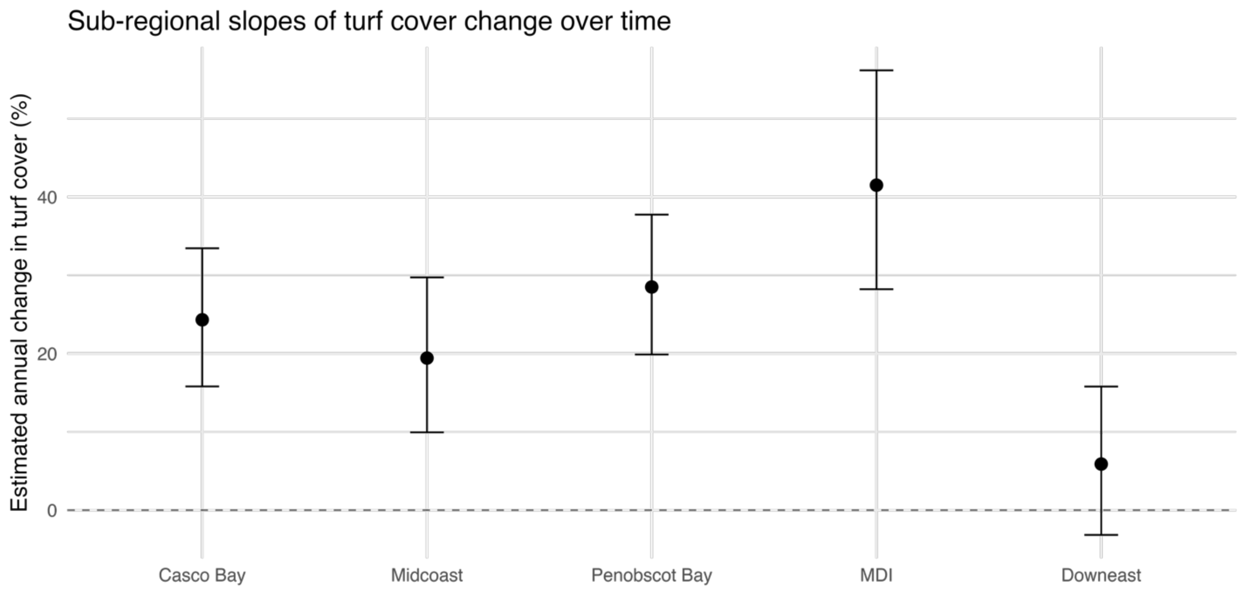

Figure S3. Relationship between kelp-to-turf metric (0 = turf-dominated reef, 1 = kelp-dominated reef) and macroalgae diversity (Simpson's diversity after a Jost transformation). Points (green circles) represent site-level observations. Black line shows fitted values from a GAM holding site and year constant at reference levels. The shaded region indicates a 95% confidence interval derived from standard errors of the predictions.

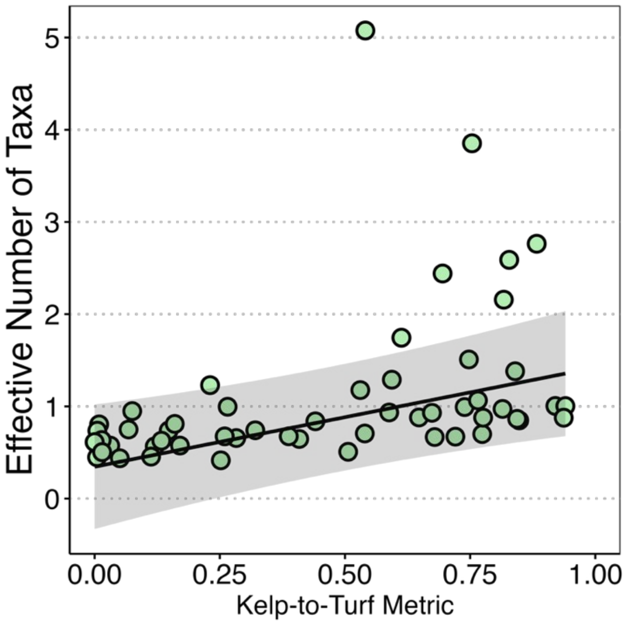

Figure S4. Piecewise structural equation model (SEM) depicting direct and indirect effects of thermal and wave conditions on turf and kelp abundance. Sea Surface Temperature Anomaly, Kelp Abundance Anomaly, and Wave Height Anomaly are annual, group mean-centered terms, while Sub-regional Sea Surface Temperature, Sub-regional Kelp, and Sub-regional Wave Height are annual, regional means. Turf Abundance (% cover) is the response. Arrow labels are standardized path coefficients from the fitted component mixed models; solid arrows indicate significant paths ($p < 0.05$) and dashed arrows indicate non-significant paths. Values below the endogenous nodes are R^2 (marginal; conditional).

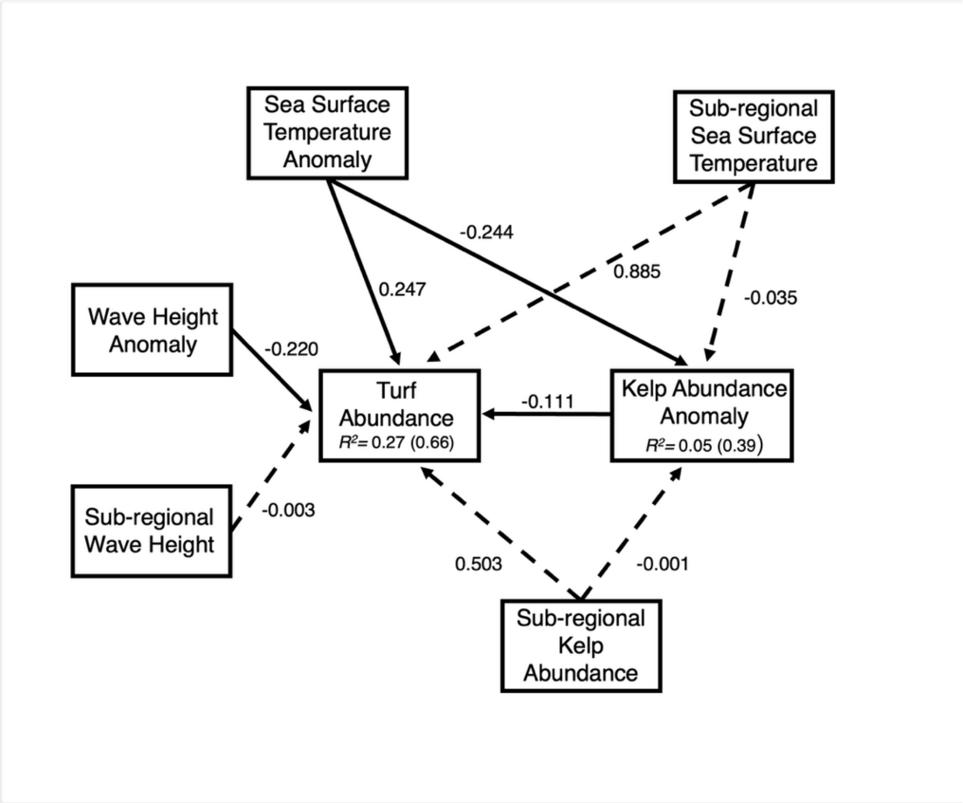

Figure S5. Relationships between kelp-to-turf metric (0 = turf-dominated reef, 1 = kelp-dominated reef) and key algal functional diversity metrics: (**A**) Functional divergence, (**B**) Functional richness, (**C**) Functional dispersion, and (**D**) Functional evenness. Points (green circles) represent site-level observations, while the black line shows fitted values from a GAM holding site and year constant at reference levels. The shaded region indicates the 95% confidence interval derived from standard errors of the predictions.

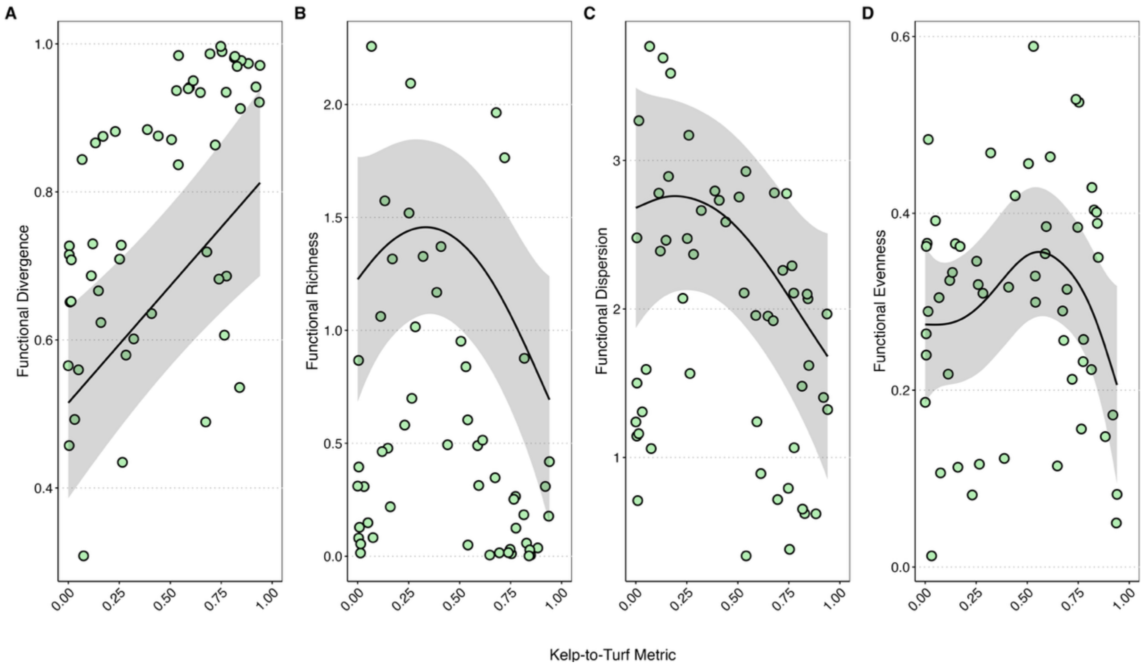

Section S1

Algal functional trait measurements

We measured surface area by spreading algae flat against a white background before taking a photo of individual algae and then analyzing each using the FIJI image processing package (Schindelin et al., 2012). We measured algal thickness with calipers for larger tubed algae and took cross-sections for all others and measured each in FIJI. We measured algal volume via water displacement in a graduated cylinder. Finally, we spun algae to remove water, weighed them (wet weight), and placed them in a 60 °C drying oven for 48 hours and reweighed them to measure dry weight. We obtained C:N ratios (alongside $\delta^{13}C$ and $\delta^{15}N$ measurements, which are not reported here) by packing ~3 mg of freeze-dried seaweed into tin capsules. Carbon and nitrogen in each sample were measured with a Costech 4010 elemental analyzer coupled to a Thermo Scientific Delta V Plus isotope ratio mass spectrometer (EA-IRMS) at the University of New Mexico Center for Stable Isotopes (Albuquerque, NM). The internationally accepted standards are Vienna Pee Dee Belemnite for $\delta^{13}C$ and atmospheric N_2 for $\delta^{15}N$. The isotopic values of our samples were corrected and calibrated to these international standards based on the analysis of in- house reference materials (green chile and blue gramma).

References

Mauffrey, A. R. L., Cappelatti, L., & Griffin, J. N. 2020. "Seaweed functional diversity revisited: Confronting traditional groups with quantitative traits." *Journal of Ecology* 108: 2390–2405.

Schindelin, J., Arganda-Carreras, I., Frise, E., Kaynig, V., Longair, M., Pietzsch, T., Preibisch, S., Rueden, C., Saalfeld, S., Schmid, B., Tinevez, J.-Y., White, D. J., Hartenstein, V., Eliceiri, K., Tomancak, P., & Cardona, A. 2012. "Fiji: An open-source platform for biological-image analysis." *Nature Methods* 9: 676-682.
